# Emerging Role of Ferroptosis in Diabetic Kidney Disease: Molecular Mechanisms and Therapeutic Opportunities

**DOI:** 10.7150/ijbs.81892

**Published:** 2023-05-11

**Authors:** Hui Wang, Dongwei Liu, Bin Zheng, Yang Yang, Yingjin Qiao, Shiyang Li, Shaokang Pan, Yong Liu, Qi Feng, Zhangsuo Liu

**Affiliations:** 1 Research Institute of Nephrology, Zhengzhou University, the First Affiliated Hospital of Zhengzhou University, Zhengzhou 450052, P. R. China.; 2 Traditional Chinese Medicine Integrated Department of Nephrology, the First Affiliated Hospital of Zhengzhou University, Zhengzhou 450052, P. R. China.; 3 Henan Province Research Center for Kidney Disease, Zhengzhou 450052, P. R. China.; 4 Key Laboratory of Precision Diagnosis and Treatment for Chronic Kidney Disease in Henan Province, Zhengzhou 450052, P. R. China.; 5 Clinical Systems Biology Laboratories, the First Affiliated Hospital of Zhengzhou University, Zhengzhou, 450052, P. R. China.; 6 Blood Purification Center, the First Affiliated Hospital of Zhengzhou University, Zhengzhou 450052, P. R. China.

**Keywords:** ferroptosis, diabetic kidney disease (DKD), regulators, molecular mechanisms, treatment progress

## Abstract

Diabetic kidney disease (DKD) is one of the most common and severe microvascular complications of diabetes mellitus (DM), and has become the leading cause of end-stage renal disease (ESRD) worldwide. Although the exact pathogenic mechanism of DKD is still unclear, programmed cell death has been demonstrated to participate in the occurrence and development of diabetic kidney injury, including ferroptosis. Ferroptosis, an iron-dependent form of cell death driven by lipid peroxidation, has been identified to play a vital role in the development and therapeutic responses of a variety of kidney diseases, such as acute kidney injury (AKI), renal cell carcinoma and DKD. In the past two years, ferroptosis has been well investigated in DKD patients and animal models, but the specific mechanisms and therapeutic effects have not been fully revealed. Herein, we reviewed the regulatory mechanisms of ferroptosis, summarized the recent findings associated with the involvement of ferroptosis in DKD, and discussed the potential of ferroptosis as a promising target for DKD treatment, thereby providing a valuable reference for basic study and clinical therapy of DKD.

## Introduction

In recent years, the number of individuals with diabetes kidney disease (DKD) is growing as the prevalence of diabetes mellitus (DM) has increased^1^. As the most common microvascular complication of DM, DKD also has a poor prognosis and limited treatment methods. According to epidemiological statistics, DKD is the primary cause of end-stage renal disease (ESRD) around the world^2^. At present, clinical treatment options for DKD mainly include angiotensin converting enzyme inhibitors (ACEIs), angiotensin II receptor antagonists (ARBs) and sodium-dependent glucose transporter 2 (SGLT2) inhibitors^3^, ^4^. Although the above therapies can slow the progression of DKD to some degree, the incidence of DKD has remained greatly high in recent years with serious consequences and economic burdens. Studies have confirmed that multiple pathophysiological disturbances contribute to the onset and development of DKD, such as hemodynamic abnormalities, oxidative stress, cell death, and genetic and epigenetic regulation 4-6. On the basis of the complicated pathogenic mechanisms and unsatisfactory therapeutic outcome of DKD, determining the exact pathogenesis and developing appropriate treatment methods are quite necessary. However, increasing evidence has considered it to be the combined result of multiple factors, and cell death attracts much attention and is deemed the direct factor affecting diabetic kidney damage^7^-^9^. Ferroptosis has recently been shown to act a crucial role in the initiation and progression of DKD^8^.

The concept of ferroptosis was firstly proposed by Dixon et al., who verified its existence in cancer cells induced by erastin^10^. Unlike other forms of regulated nonapoptotic cell death, ferroptosis is dependent on iron overload and characterized by the accretion of lipid peroxides^10^, ^11^. The morphologic traits of ferroptosis include cell membrane rupture and blistering, mitochondrial shrinkage, and the reduction or vanishing of mitochondrial cristae. Biochemically, the deposition of intracellular iron, abundance of reactive oxygen species (ROS) and aggregation of lipid peroxides are manifested during the occurrence of ferroptosis^12^. With more advanced research, ferroptosis was discovered to be pivotal in a range of diseases, including cancer, aging, neurological diseases, cardiovascular diseases and kidney diseases^13^-^15^. In kidney diseases, most scientists have paid more attention to the correlation of ferroptosis and acute kidney injury (AKI), renal cell carcinoma and polycystic kidney 15-17, and relatively little concrete evidence to support ferroptotic contribution to the development of DKD. In this review, we systematically discuss the research progression of ferroptosis in DKD, which may provide valuable references for elucidating the pathogenesis of DKD and thereby creating targeted remedy to ferroptosis.

## Key mechanisms of ferroptosis

**Figure 1:**
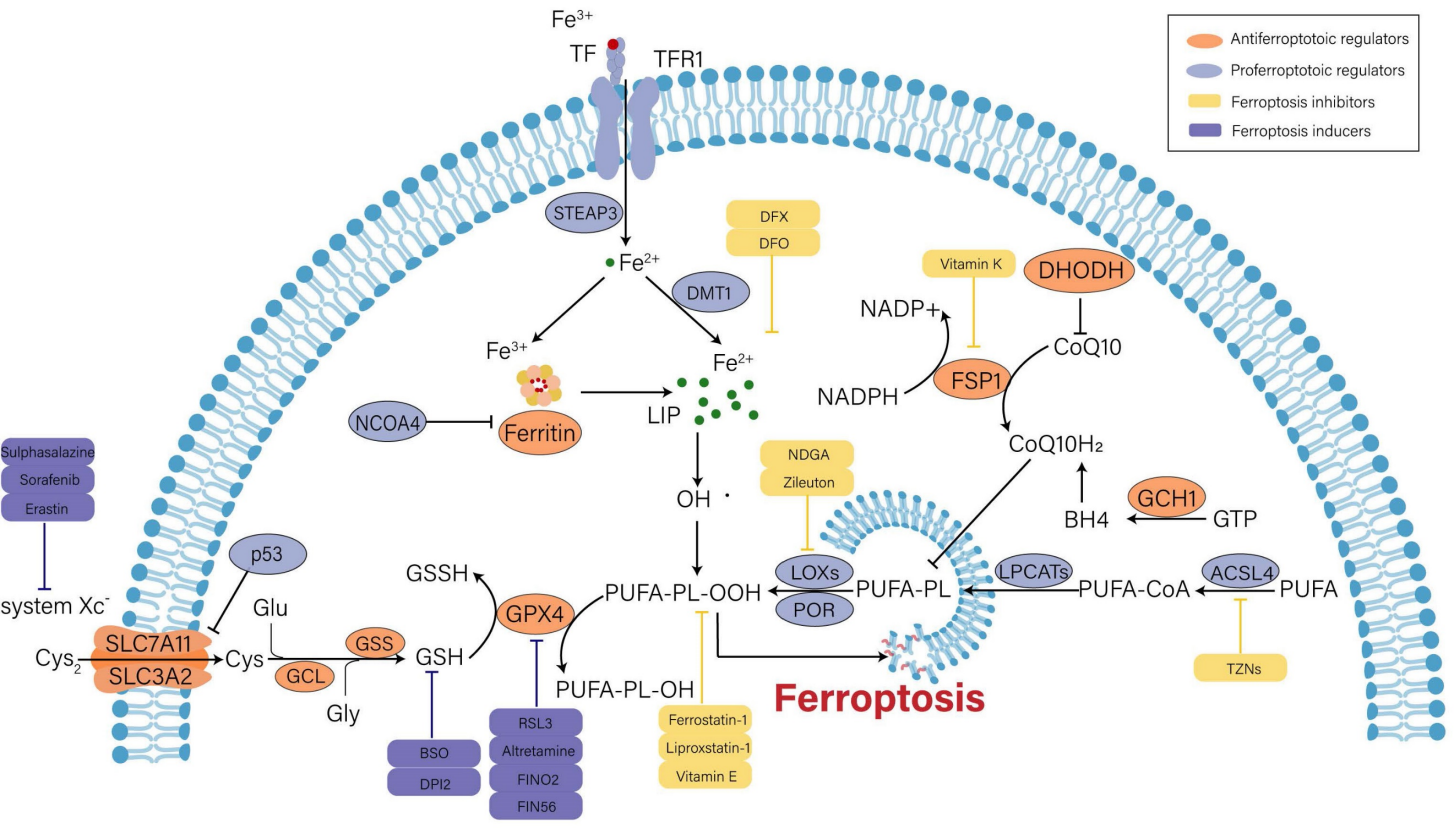
** Mechanisms and major regulators of ferroptosis.** Iron metabolism disorder, lipid peroxidation and decreased antioxidant capacity are involved in the occurrence of ferroptosis, and several key regulators (e.g., system Xc^-^, GPX4, p53 and ACSL4) play an important role in monitoring ferroptosis. Some recently developed compounds can induce or inhibit ferroptosis by targeting these key regulators.

Over the past decade, the understanding of ferroptotic mechanisms has made rapid progress. Beginning with the discovery of the part of cysteine-glutathione (GSH)-glutathione peroxidase 4 (GPX4) signaling in inhibition of ferroptosis, the executioner position of lipid peroxidation in ferroptosis is now convinced. Moreover, GPX4-independent ferroptosis surveillance mechanisms have been disclosed. Herein, we will briefly illustrate the major mechanisms in the occurrence of ferroptosis (**Figure 1**).

### Iron metabolism disorder

Under general physiological conditions, circulating ferric iron (Fe^3+^) binds to transferrin (TF) and is transported into cells intermediated by the membrane protein transferrin receptor 1 (TFR1). Subsequently, Fe^3+^ is reduced into ferrous iron (Fe^2+^) by six-transmembrane epithelial antigen of prostate 3 (STEAP3). Divalent metal transporter 1 (DMT1) regulates the release of Fe^2+^ into the labile iron pool (LIP) for further utilization, and extra Fe^2+^ is retained in ferritin. Ferritin is composed of ferritin light chain (FTL) and ferritin heavy chain 1 (FTH1), and it can catalyze Fe^2+^ conversion to Fe^3+^ to maintain iron homeostasis in the cytoplasm 18, 19. On the one hand, excess free iron can produce hydroxyl radicals when it reacts with hydrogen peroxide (H_2_O_2_). Oxidative stress is caused by an imbalance in the rate of free radical formation and detoxification, which ultimately triggers ferroptosis via lipid peroxidation^20^. On the other hand, surplus Fe^2+^ induces the activation of ferric enzymes such as lipoxygenases (LOXs) and further promotes lipid peroxidation in cytomembrane, which eventually results in cell death^21^. Therefore, enhanced iron uptake and decreased iron output will increase the vulnerability of cells to oxidative harm and ferroptosis. Ferritinophagy is defined as the selective autophagic degradation of ferritin, which prompts the accumulation of cytosolic Fe^2+^. Nuclear receptor coactivator 4 (NCOA4) is the crucial regulator of ferritinophagy^22^. In renal tubular epithelial cells (RTECs), knockdown of NCOA4 could ameliorate ferroptosis caused by the activation of autophagy^23^.

### Lipid peroxides accumulation

Lipid peroxidation is viewed as an initiation signal of ferroptosis^24^. Compared with unsaturated fatty acids and monounsaturated fatty acids, polyunsaturated fatty acids (PUFAs) are more prone to lipid peroxidation due to unstable carbon‒carbon double bonds and contribute to ferroptosis^25^. Free PUFAs are esterified by activated acyl-CoA synthetase long chain family member 4 (ACSL4) and transferred to membrane phospholipids by lysophosphatidylcholine acyltransferase 3 (LPCAT3). Then, they are oxidized to toxic lipid peroxides by LOXs. Among PUFAs-related phospholipids, phosphatidylethanolamines (PEs) of arachidonic acid (AA) and adrenic acid (AdA) are the main substrates during the process of lipid peroxidation^24^. The severity of ferroptosis depends on the aggregation degree of PUFAs. Additionally, the activity of ACSL4 and LOXs is absolutely indispensable for ferroptosis execution. Doll et al. argued that ACSL4 guided ferroptotic cell death by remodeling the cellular lipid composition^26^. Inhibitors of LOXs, such as liproxstatin-1 and vitamin E, are effective for preventing ferroptosis^27^.

Recent studies discovered that cytochrome P450 oxidoreductase (POR) was also implicated in driving lipid peroxidation^28^, ^29^. POR is widely expressed in the endoplasmic reticulum and donates electrons from nicotinamide adenine dinucleotide phosphate (NADPH) to cytochrome P450 (CYPs) and other proteins^30^. It might initiate lipid peroxidation by facilitating Fenton reactions in the heme component of CYPs^28^. Nevertheless, another study stated that the interaction between POR and its downstream electron receptors was not required for ferroptosis induction, and POR was sufficient to independently impair PUFA-containing phospholipid membranes^29^. Thus, the role of POR in ferroptosis cannot be completely concluded and remains to be further investigated.

### Antioxidant capacity imbalance

Under normal conditions, cells exchange intracellular glutamate (Glu) and extracellular cystine (Cys_2_) at a ratio of 1:1 through the cystine/glutamate anti transporter (system Xc^-^). System Xc^-^, a disulfide heterodimer consisting of light chain subunits (SLC7A11) and heavy chain subunits (SLC3A2), is extensively distributed in cellular membranes^10^. Once in cells, Cys_2_ can be reduced to cysteine (Cys) for the following synthesis of GSH, which is catalyzed by glutamate cysteine ligase (GCL) and glutathione synthetase (GSS)^11^. GSH is an intracellular antioxidant that participates in enzymatic and nonenzymatic reactions to maintain the normal level of hydrogen peroxide in cells. GPX4 can reduce lipid hydrogen peroxide (L-OOH) to nontoxic lipid hydroxyl derivatives (L-OH) in a GSH-dependent manner, so as to interrupt ferroptotic cell death caused by harmful lipid peroxides^31^. Therefore, intracellular GSH depletion or GPX4 inactivation induced by various factors will decrease the clearance rate of toxic lipid peroxides and result in ferroptosis. For example, erastin can inhibit cystine uptake and synthesis of GSH by blocking system Xc^-^, thereby promoting ferroptosis progression^10^. RSL3 induces ferroptosis by inactivating GPX4 activity^32^. In addition, it was proven that inducible knockout of GPX4 could make acute kidney failure through the ferroptotic pathway 33.

Undoubtedly, GPX4 is the core regulator of ferroptosis. However, three pathways independent of GPX4 have been identified in the past few years, including ferroptosis suppressor protein 1 (FSP1)/coenzyme Q10 (CoQ10), GTP cyclohydrolase 1 (GCH1) and dihydroorotate dehydrogenase (DHODH). FSP1 can regenerate the reduced form of CoQ10 (CoQ10H_2_) utilizing NADPH. CoQ10 eventually removes lipid peroxides through an oxidation reaction^34^. GCH1 suppresses ferroptosis mainly by generating the lipophilic antioxidant tetrahydrobiopterin (BH4) and remodeling the lipid membrane environment to increase CoQ10H_2_ and deplete PUFAs^35^. Moreover, DHODH was found to inhibit ferroptosis by reducing mitochondrial CoQ10^36^.

### Other regulators

Beyond the typical regulatory pathways mentioned above, ferroptosis is modulated by many regulators, such as p53, nuclear factor erythroid 2-related factor 2 (Nrf2) and AMP-activated protein kinase (AMPK)^37^-^39^, among which p53 gets a lot of attention due to its controversial role in ferroptosis.

The effect of p53 on ferroptosis is double-sided^37^. It was reported that p53 could promote ferroptosis by repressing the expression of SLC7A11 in both transcriptional and non-transcriptional manners, which led to the decreased uptake of Cys2 and impaired GSH biogenesis^40^, ^41^. Additionally, p53 could transactive spermidine/spermine N1-acetyltransferase 1 (SAT1), a rate-limiting enzyme in polyamine catabolism. SAT1 was demonstrated to induce ferroptosis by elevating the expression of arachidonate 15-lipoxygenase (ALOX15)^42^. Chu et al. identified that ALOX12-mediated lipid peroxidation was engaged in p53-dependent ferroptotic responses^43^. Moreover, glutaminase2 (GLS2), cytochrome c oxidase 2 (SCO2) and several noncoding RNAs were confirmed to be the target molecules of p53-mediated ferroptosis 44, 45. However, p53 seems to retard ferroptosis in certain cellular metabolic environments. A study proved that p53 could bind to dipeptidyl-peptidase 4 (DPP4) to reduce the DPP4-dependent oxidization of PUFAs to inhibit ferroptosis in colorectal cancer cells^46^. Upregulation of cyclin dependent kinase inhibitor 1A(CDKN1A)/p21 by p53 contributed to the reduced sensitivity to ferroptosis in cancer cells. The underlying mechanism might be the p21-induced increase in GSH synthesis^47^. Other regulatory factors will be described in the following sections.

## Advances in the pharmacology of ferroptosis

Given the multiple regulators in the occurrence of ferroptosis, targeting certain pathways can induce or inhibit this process. Ferroptosis induction can be applied to eliminate cancer cells, and suppression of ferroptosis can be a potent therapy in other diseases, such as cardiovascular disease, neurodegenerative diseases and DKD^12^. In the past decade, an increasing number of small molecules have been verified to be valuable in regulating ferroptosis, as detailed in **Table 1**.

## Research progress of ferroptosis in DKD

Previous studies have found that iron overload was directly associated with proteinuria and tubular damage in DKD patients and streptozotocin (STZ)-induced rat models of type 1 diabetes 78, 79. Excess iron content was detected in lysosomes of proximal tubule epithelial cells, which could induce renal dysfunction in DKD patients and mice^80^. In diabetic rats, a low-iron diet or being fed with iron chelators could delay the development of DKD, indicating that iron overload could aggravate kidney injury in DKD^81^. Investigations also proved that the transferrin level and iron content in RTECs of DKD patients are higher than those in healthy individuals^82^. Overall, these findings reveal that the development of DKD is inseparable from iron overload.

Nowadays, numerous studies have proven that ferroptosis occurs in RTECs in diabetic animal models and prompts kidney function deterioration. Wang et al. confirmed that ferroptosis was involved in kidney injury in STZ-induced type 1 diabetic mice and *db/db* mice^68^. Afterward, Kim et al. found that the expression levels of SLC7A11 and GPX4 were significantly downregulated in renal biopsy samples of diabetic patients. Meanwhile, their experiments also indicated that the levels of ferroptosis-associated biomarkers such as iron content and toxic lipid peroxides were increased in transforming growth factor beta1 (TGF-β1)-stimulated NRK-52E cells and type 1 diabetic mice^83^. In addition to RTECs, ferroptosis can be seen in almost all types of renal cells in DKD. Zhang et al. argued that mitigation of ferroptosis can prevent high glucose-induced podocyte injury^84^. High fructose could also induce podocyte ferroptosis and ultimately cause glomerular impairment^85^. In mesangial cells, molecules related to ferroptosis are altered under high glucose condition, which can be antagonized by deferoxamine, a ferroptosis inhibitor^86^. In addition, ferroptosis is instrumental in the process of endothelial dysfunction caused by hyperglycemia^87^. Therefore, evidence is mounting that ferroptosis does prompt the development of DKD, and inhibition of ferroptosis may be a therapeutic option for DKD.

## The crosstalk between ferroptosis and other cellular processes in DKD

**Figure 2:**
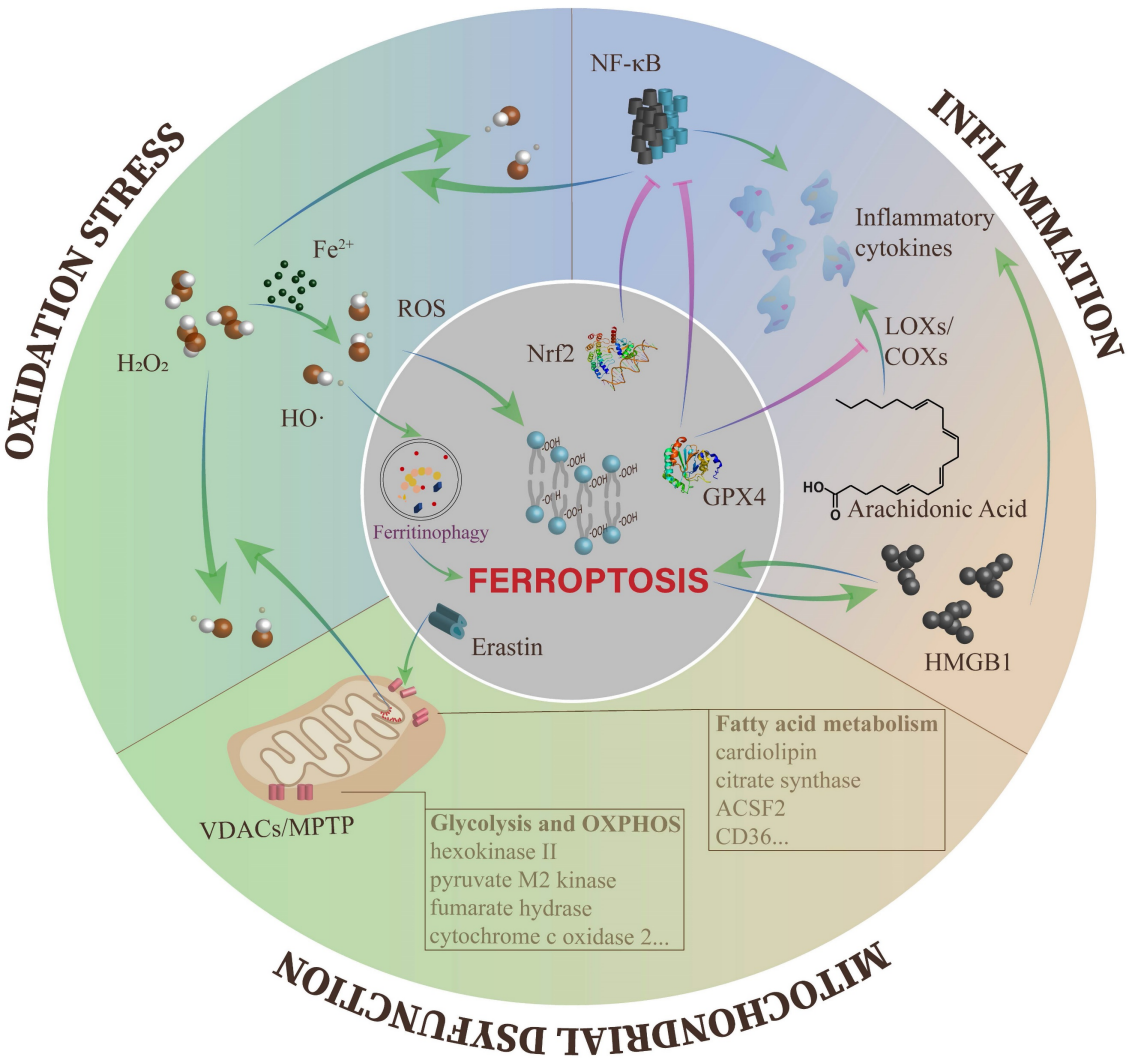
** The crosstalk between ferroptosis and other cellular processes of DKD.** The overproduction of ROS leads to different kinds of downstream impairments, including ferroptosis. Meanwhile, the Fenton reaction containing excess free iron is one of the main pathways to produce ROS. Ferroptosis and inflammation can affect each other and tend to form an autoamplification loop. In mitochondria, the opening of VDACs plays a key role in mitochondrial dysfunction during ferroptosis, and multiple metabolic enzymes are influenced by ferroptosis.

With deepening research on ferroptosis in various diseases, increasingly regulatory mechanisms of ferroptosis have been discovered^14^, ^15^, ^50^. More importantly, several classical regulators of DKD have been identified to effectively modulate ferroptosis^83^, ^88^. Herein, we will describe the interactions between ferroptosis and the typical pathogenesis of DKD (**Figure 2**).

### Ferroptosis and oxidative stress

In DKD, almost all pathogenesis is inseparable from the overactivation of ROS, which is the connection point between metabolism abnormalities and hemodynamic changes^89^. ROS accumulation can be disruptive to all types of kidney cells at the gene, transcription and protein levels, eventually resulting in inflammation, fibrosis and endothelial dysfunction^90^-^92^.

Along with the decline in cellular antioxidant capacity, there are many ways to produce ROS in DKD. In consideration of the surplus iron in DKD, the Fenton reaction is an essential part of ROS generation^89^. In ferroptosis, the most direct pathogenic mechanism caused by ROS is lipid peroxidation, which ultimately triggers ferroptotic cell death^49^. In addition, ROS can be involved in erastin-induced ferroptosis by ferritin autophagy to upregulate intracellular available iron levels^93^. In recent time, Wang et al. found close relationships between GPX4 and DKD, including urinary protein, Scr, eGFR, and the percentage of sclerosed glomeruli in renal specimens. They also proposed that the GPX4 level in the tubulointerstitium was an independent predictor for renal outcomes^94^.

To sum up, ROS act as a junction in the action of DKD-induced ferroptosis. As a result, using antioxidants (e.g., quercetin and vitamin E) was considered as a clinical therapy for DKD patients, but the trials are still at the stage of animal studies^95^, ^96^. Given the effect of ROS on ferroptosis, it is possible to develop effective drugs to modulate ferroptosis in DKD.

### Ferroptosis and inflammation

Abundant evidence indicates that inflammation is important in the progression of DKD^97^. In DKD, hyperglycemia induces the overexpression of chemokines and cytokines in damaged glomeruli and renal tubules, which aggravate renal injury through a variety of mechanisms, such as leukocyte infiltration, renal filtration barrier breakdown and mesangial proliferation^97^, ^98^. These proinflammatory molecules also induce the above lesions through the nuclear factor-κB (NF-κB), Janus kinase/signal transducers and activators of transcription (JAK/STAT), and TGF-β/Smad signaling pathways^99^-^101^.

Inflammation can be inhibited by negative regulators of ferroptosis. AA is a precursor of bioactive proinflammatory mediators. During the catalytic process, the level of cellular lipid peroxides directly affects the activity of enzymes, including cyclooxygenases (COXs) and LOXs. LOXs activation can also be blocked when the cell is in a redox state, such as the activation of GPX4^102^. GPX4 activation can also reduce ROS levels by suppressing NF-κB, thus effectively reducing cell damage induced by inflammation^103^. In addition, Nrf2 can inhibit the transcription of NF-κB^104^ and the encoding of proinflammatory genes, including interleukin 6 (IL-6) and IL-1β^105^. In an oxalate-induced AKI mouse model, ferrostatin-1 mitigated the infiltration of neutrophils and the expression of proinflammatory cytokines, including chemokine ligand 2 (CXCL-2) and IL-6^106^. In fact, ferroptosis can induce inflammation by releasing IL-33 and other unidentified pathways^11^. Ferroptotic cells can release high mobility group 1 (HMGB1) in an autophagy-dependent manner. As a damage-associated molecular pattern (DAMP), HMGB1 activates immune cells to amplify inflammation through the release of multiple cytokines or chemokines^107^.

Conversely, several inflammatory cytokines, such as prostaglandin E2 (PGE2), IL-1β and IL-6, have been confirmed to directly affect the onset of ferroptosis^87^, ^108^, ^109^. HMGB1 can regulate ferroptosis in response to high glucose in DKD^86^. LOXs also activate immune cells through LOX-derived proinflammatory metabolites, including leukotriene B4 (LTB4), LTC4, LTD4, LTE4 and Hydroxyeicosatetraenoic acids (HETEs)^110^, which indirectly lead to ferroptosis.

According to mounting evidence, the onset of ferroptosis is always accompanied by the presence of inflammation. More intriguingly, the cross-link between ferroptosis and inflammation is mutually induced and drives a local autoamplification loop^60^. The internal relationship between ferroptosis and aseptic inflammation has been valued by increasing researchers and has provided a direction for unraveling the relationship between ferroptosis and DKD.

### Ferroptosis and mitochondrial dysfunction

Mitochondria are the core organelles of cellular energy metabolism^111^. Hyperglycemia results in the storage of metabolic substrates, causing the accumulation of glycolytic intermediates and the formation of mitochondrial ROS (mtROS) from the electron transport chain (ETC), ultimately inducing mitochondrial dysfunction and energy metabolism imbalance^112^, ^113^.

Mitochondrial homeostasis is pivotal in maintaining normal kidney functions, and the typical morphological feature of ferroptosis is mitochondrial damage. However, what causes irreversible mitochondrial damage during ferroptosis? Studies suggest that erastin and its analogs antagonize the functions of free tubulin on voltage-dependent anion channel 2/3 (VDACs) and induce the opening of VDACs. VDACs contribute to regulating the passive diffusion of anionic hydrophilic mitochondrial metabolites^114^. Subsequently, with the increase in mitochondrial membrane potential, the mitochondria are depolarized, and large amounts of mtROS are generated^115^, ^116^. In addition, glutamate can enhance the production of mtROS through opening of the mitochondrial permeability transition pore (MPTP) and trigger dissipation of the mitochondrial transmembrane potential and depletion of ATP 117, 118. The opening of VDACs and excessive ROS production cause mitochondrial Ca^2+^ overload, which ultimately induces mitochondrial dysfunction and the loss of mitochondrial membrane integrity^119^.

A reduction in glycolytic progress was detected in erastin-induced ferroptosis, which might be caused by increased synthesis of ATP and oxidative phosphorylation (OXPHOS)^10^, ^115^. Some crucial enzymes of glycolysis were found to be decreased in cancer cells treated with RSL3, including hexokinase II and pyruvate M2 kinase^120^. In addition, several enzymes engaged in mitochondrial respiration are found to regulate ferroptotic cell death, such as SCO2 and fumarate hydrase (FH)^44^, ^116^. However, the enzymes mentioned above all need to be examined in DKD models.

Moreover, fatty acid (FA) metabolism in mitochondria has a close relationship with lipid oxidation in ferroptosis. Citrate synthase (CS) and acyl-CoA synthetase family member 2 (ACSF2), which regulate FA activation and synthesis respectively, were identified to be highly correlated with erastin-induced ferroptosis^10^. In addition, large amounts of the mitochondria-specific phospholipid cardiolipin (CL) were administered by oxidative modification in *GPX4^-/-^* kidneys^33^. Recently, FA in the tumor microenvironment has been shown to trigger CD8^+^ T cell ferroptosis in a CD36-dependent manner^121^. CD36 is the main pathway of FA uptake and is highly expressed in all kidney cells^122^. In RTECs cultured with high glucose medium, they found increased expression of CD36 and enlarged uptake of FA mediated by CD36^123^, ^124^. Therefore, it is also worth investigating whether CD36 is related to ferroptosis in DKD patients.

## Ferroptosis-related regulators involved in DKD

In DKD, the key regulators of ferroptosis involved in typical metabolic pathways illustrated above have been broadly explored. Therefore, in this section, we will introduce the emerging molecular mechanisms of ferroptosis regulation in DKD.

### Nuclear factor erythroid 2-related factor 2 (Nrf2)

Nrf2 is a transcription factor of the basic region leucine zipper family^125^. Previous studies have demonstrated that Nrf2 plays a crucial role in responding to antioxidative stress and maintaining redox homeostasis. In the mechanism of DKD, in addition to Kelch-1ike ECH-associated protein l (Keap1)/Nrf2/antioxidant response element (ARE) signaling, Nrf2 can inhibit inflammation to relieve kidney damage by suppressing NF-κB, Sirtuin1 (SIRT1) and nucleotide-binding oligomerization domain-like receptor protein 3 (NLRP3)^104^, ^126^. Controversially, Nrf2 seems to have a dual effect in DKD. Zhao et al. elucidated that Nrf2 deficiency attenuated kidney injury by upregulating the expression of intrarenal angiotensin-converting enzyme-2 (ACE2) and angiotensin 1-7 receptor in DKD mice^127^. Therefore, the role of Nrf2 in DKD should be further discussed.

Nowadays, researchers have paid more attention to the connection between Nrf2 and ferroptosis. Activated Nrf2 was firstly found to inhibit ferroptosis by regulating the expression of NAD(P)H quinone oxidoreductase 1 (NQO1), heme oxygenase-1 (HO-1) and FTH1 in hepatocellular carcinoma^50^. Then, multiple studies have confirmed that Nrf2 can regulate a host of genes associated with ferroptosis, such as ferroportin (FPN), GPX4, small heterodimer partner (SHP) and peroxisome proliferator-activated receptor γ (PPARγ)^50^, ^128^. In summary, Nrf2 has been found to regulate ferroptosis through three pathways, including iron metabolism^129^, lipid metabolism^39^ and intermediary metabolism^130^. Therefore, Nrf2 is identified as an important ferroptosis regulator and an attractive pharmacological target in DKD. Kota et al. found that blocking activin receptor-like kinase 4/5 (ALK4/5, one of the TGF-β receptors) could activate Nrf2 signaling to inhibit erastin-induced ferroptosis in HK-2 cells^131^. In NRK-52E cells, TGF-β1 stimulation considerably upregulated the expression levels of p53 and nuclear Nrf2 protein, which was consistent with the trend of erastin-induced cells and animal models, indicating that Nrf2 is probably related to ferroptosis under diabetic conditions^83^. Activation of the Nrf2/FPN pathway could block ferroptosis and thus reduce myocardial ischemia-reperfusion injury (IRI) in diabetic rats^132^. Inhibition of Nrf2 enhanced the sensitivity of RTECs to ferroptosis caused by high glucose, and upregulating Nrf2 reversed the expression of ferroptosis-related proteins to ameliorate cell injury under high glucose conditions^88^. In mesangial cells, HMGB1 can regulate ferroptosis through the Nrf2 pathway under high glucose conditions^86^. Hence, the effect of Nrf2 on ferroptosis involved in DKD is indispensable.

### Heme Oxygenase-1 (HO-1)

HO-1 catalyzes the rate-limiting step in the catabolism of heme into biliverdin, carbon monoxide (CO) and iron. Numerous studies have demonstrated that HO-1/biliverdin/CO is involved in the progression of anti-inflammatory and antioxidant stress^133^. In DKD, the high level of HO-1 induced by Nrf2 or hypoxia inducible factor-1α (HIF-1α) effectively decreased ROS generation and alleviated kidney injury^133^, ^134^. Studies have found that ferroptosis inducers upregulated the expression of HO-1 in RTECs^135^. However, it is not clear whether the increased HO-1 is induced as a protective response or accelerates ferroptosis. Feng et al. proposed that renal tubular damage and fibrosis in *db/db* mice were associated with elevated levels of HIF-1α and HO-1, which led to iron accumulation in renal tubules by downregulation of ferritin. Inhibition of HO-1 mitigated iron accumulation, thereby preventing lipid peroxidation by reducing ROS production^136^. However, in another study, HO-1 was found to suppress erastin-induced ferroptosis of RTECs^135^. Interestingly, in a contrast-induced nephropathy (CIN) model, type 1 diabetic rats received intravenous injection of iopromide, heme inhibited ferroptosis by activating HO-1/Nrf2 and upregulating GPX4. They suggested that HO-1 and Nrf2 constitute a positive mutually reinforcing cycle, resulting in self-reinforcement and maintenance of antioxidant capacity^137^. To date, the controversial points of HO-1 in ferroptosis might be dependent on the comprehensive regulation of iron content and antioxidant activity. It is challenging that HO-1 produces a cytoprotective effect against kidney injury in DKD. Therefore, more research is necessary to determine the actual function of HO-1 in DKD.

### High Mobility Group Box-1 (HMGB1)

HMGB1 is a ubiquitously expressed nuclear protein engaged in the progression of nucleosome stabilization and DNA binding. It has been found to act as a proinflammatory cytokine in many pathologies, such as sepsis, IRI and cancer^138^. Interrupting the contact between HMGB1 and its receptor was shown to effectively prevent the occurrence and development of DKD^139^. Additionally, HMGB1 was described as a new mediator of ferroptosis by regulating the rat sarcoma (RAS)/c-Jun N-terminal kinase (JNK)/p38 signaling pathway and a potential target for leukemia therapy^140^. A recent study reported that suppression of HMGB1 could alleviate high glucose-induced ferroptosis in glomerular mesangial cells and ameliorate the progression of inflammation. They also found that HMGB1 knockdown inhibited the high glucose-induced toll-like receptor 4 (TLR4)/NF-κB signaling pathway and promoted the expression of Nrf2 and its downstream targets, such as HO-1, NQO1, glutamate-cysteine ligase catalytic subunit (GCLC) and modifier subunit of glutamate cysteine ligase (GCLM)^86^. Taken together, it can be concluded that HMGB1 is of great importance in ferroptosis regulation during the initiation and progression of DKD and seems to be an unneglected point of intersection between ferroptosis and inflammation in DKD.

### ZRT/IRT-like protein 14 (ZIP14)

ZIP14 is a divalent iron import protein that is distributed in both the proximal and distal tubules, and mainly takes up nontransferrin-bound iron from the lumen^141^, ^142^. Iron accumulation can increase lipid deposition in intestinal epithelial cells. Notably, the upregulation of ZIP14 and FPN acts a significant role in this process^143^. Increased ZIP14 in proximal and distal tubules has been observed in focal segmental glomerulosclerosis and IgA nephropathy, which will aggravate cell oxidative damage 141. Inhibition of ZIP14 in liver TFR-specific knockout mice ameliorated ferroptosis-mediated liver fibrosis^144^. Wu et al. demonstrated that ZIP14 might be involved in iron deposition and trigger ferroptosis in DKD rats, and inhibition of ZIP14 expression in HK-2 cells could suppress high glucose-induced ferroptosis^145^.

### N-acetylcysteine (NAC)

N-acetylcysteine (NAC) is a sulfhydryl compound that is an acetylated form of L-cysteine. NAC is a precursor of reduced glutathione, which exhibits direct or indirect antioxidant properties^146^, ^147^. Recent findings demonstrated that NAC exerted protective effects against diabetic kidney injury by inhibiting oxidative stress^147^. In a study on myocardial IRI in diabetic rats, NAC alleviated ferroptotic cell death and oxidative injury^148^. A recent investigation stated that combination therapy with NAC and insulin alleviated kidney tissue damage by suppressing ferroptosis in a diabetic beagle dog model induced by STZ and alloxan (ALX). Further experiments confirmed that NAC mainly increased the expression of GPX4 by upregulating sirtuin-3 (SIRT3) and inhibiting the acetylation of superoxide dismutase 2 (SOD2), thereby inhibiting cell ferroptosis and improving kidney function in DKD^149^.

### AMP-activated protein kinase (AMPK)

AMPK, as a central regulator of energy homeostasis, has been widely investigated in diverse human diseases and is considered a promising interference target in DKD. It was observed that the expression of AMPK is downregulated in the renal tissue of high-fat diet (HFD)-induced obese rats^150^. A recent study found that under conditions of energy stress, such as glucose starvation and ATP depletion, activated AMPK is crucial for inhibiting erastin-induced ferroptotic cell death^38^. It was proven that AMPK inhibited PUFAs synthesis by inhibiting acetyl-CoA carboxylase (ACC) activity. Activated ACC catalyzes the synthesis of malonyl-CoA from acetyl-CoA, the rate-limiting step in FA synthesis. Similarly, the function of AMPK in ferroptosis has been detected in cardiomyocytes through another pathway. In this study, AMPK was verified to inhibit ferroptosis induced by IRI in diabetic hearts via inactivation of NADPH oxidase 2 (NOX2), suggesting that the inhibitory effect of AMPK/NOX2 on ferroptosis may be another important protective mechanism in infarcted hearts^148^. In *db/db* mice, previous studies found that AMPK activation could reduce ROS production and increase mitochondrial biogenesis and ETC activity to alleviate kidney damage in diabetic models^150^, ^151^. To date, direct evidence for the effect of AMPK on renal cell ferroptosis under diabetic conditions is not sufficient. However, it is worth investigating whether AMPK has positive impacts on DKD by inhibiting ferroptosis.

### Other regulatory factors

In addition to the regulatory mechanisms described above, several new mechanisms have been revealed recently. For example, Jin et al. found that a murine *Vezf1* gene, mmu_ circRNA_ 0000309, could upregulate GPX4 expression by competitively binding miR-188-3p, which further alleviated mitochondrial and podocyte damage by inhibiting ferroptosis. However, hsa_circ_0000309 is totally different from mmu_circ_0000309. For application to human DKD, the candidate gene needs to be discovered^152^. In type 1 diabetic mice and high glucose-cultured HK-2 cells, it was demonstrated that circ_ASAP2 binding to miR-770-5p reduced inflammation and ferroptosis by regulating the SRY-box transcription factor 2 (SOX2)/SLC7A11 pathway^153^. Peroxiredoxin-6 (Prdx6), a new member of the antioxidant enzyme family, is a negative mediator of ferroptosis^154^. Some experiments also confirmed that Prdx6 could moderate lipopolysaccharide (LPS)-induced AKI by decreasing oxidative stress and inflammation^155^. Zhang et al. found that the expression of Prdx6 was regulated by specificity protein 1 (Sp1) at the transcriptional level, and the upregulation of Prdx6 could prevent podocyte impairment in DKD by alleviating oxidative stress and ferroptosis^84^. Salusin-β, a bioactive peptide containing 20 amino acids, is translated from the alternatively spliced mRNA of the torsion dystonia related gene (TOR2A)^156^. Salusin-β participates in high glucose-induced ferroptosis dependent on Nrf2 in HK-2 cells^157^. A bioinformatics analysis showed that ferroptosis was involved in the significantly enriched biological processes in the diabetic tubulointerstitium^158^. Hu et al. screened hub genes based on the weighted correlation network analysis (WGCNA) algorithm and identified interconnected transcription factors (TFs) and non-coding RNAs (ncRNAs) using principal component analysis. *FPR3, C3AR1, CD14, ITGB2, RAC2 and ITGAM* are hub genes related to ferroptosis at the onset of DKD^159^. Likewise, ALOX15-mediated lipid metabolism was detected in all intrinsic glomerular cells via bioinformatics analysis. MiR-142 and miRNA-650 might be involved in regulating ALOX15^95^. Recently, urine metabolomics revealed metabolic alterations in DKD, including the ferroptosis signaling pathway^160^. An increasing number of investigations have identified genes associated with ferroptosis using bioinformatics methods, and the specific mechanism needs to be explored by more experiments.

## Pharmacological studies related to ferroptosis in DKD

Given its contribution to the pathologic process of DKD, ferroptosis is an emerging target in treating and preventing the progression of kidney damage. In this section, we aim to describe the recent progress of experimental drugs targeting ferroptosis in diabetic animal or cell models. It can be concluded that suppressing ferroptosis has great potential for the remedy of DKD (**Table 2**).

### Hypoglycemic drugs

Hypoglycemic drugs are beneficial to kidney function by decreasing albumin urine and delaying the progression of kidney fibrosis in DKD^3^. However, more and more studies have found that they can alleviate kidney dysfunction by inhibiting ferroptosis.

Thiazolidinediones (TZDs) can reduce blood glucose levels and are mainly associated with enhanced insulin sensitivity^167^. In addition, as an ACSL4 inhibitor, TZDs can diminish the plasma free FA concentration. It was reported that rosiglitazone is the strongest ACSL4 inhibitor 168. Wang et al. verified that rosiglitazone could reduce blood glucose and urinary albumin levels by inhibiting ferroptosis and inflammation in type 1 and type 2 diabetic mice^68^. Fenofibrate is involved in lipid regulation the activation of PPARs. In addition to lowering blood lipids, it can improve renal lesions and reduce proteinuria caused by hyperglycemia^169^. A recent experiment verified that fenofibrate affected ferroptosis-related signaling pathways to protect kidney function from DKD, and Nrf2 was confirmed to act a significant role in the process of mitigation^88^. SGLT-2 inhibitors reduced glucose reabsorption in proximal tubules through SGLT2 to promote a hypoglycemic effect^170^. Quagliariello et al. reported that empagliflozin (EMPA) could significantly improve cardiac function by reducing ferroptosis through NLRP3- and myeloid differentiation primary response 88 (MyD88)-related pathways in mice treated with doxorubicin 171. In addition, it was reported that dapagliflozin (DAPA) could alleviate the performance of DKD by suppressing ferroptosis. The mechanism could be that DAPA binding to FPN led to the inhibition of ubiquitination degradation of FPN^163^, which provided a new perspective for DAPA application in the treatment of DKD. Liraglutide, intrinsically a human glucagon-like peptide-1 (GLP-1) analog^172^, could activate the Nrf2/HO-1 pathway to mitigate hepatocyte fibrosis in *db/db* mice by relieving ferroptosis^173^. Besides, it could alleviate the cognitive impairment of diabetic mice by reducing ROS and iron deposition^174^. However, the role of liraglutide in DKD has not been explored.

In conclusion, it is not difficult to find that the effect of hypoglycemic drugs on hypoglycemic or renal protection is closely connected with iron metabolism, Nrf2 and lipid metabolism in addition to the classical mechanisms. Therefore, in some ways, it reflects that ferroptosis plays an important role in the progression of DKD.

### Hypotensive drugs

As a result of a decreased glomerular filtration rate, metabolic disorders and renin-angiotensin system activation, hypertension is often accompanied by DKD. Many hypotensive drugs can reduce the urinary albumin excretion rate and slow the progression of kidney diseases^4^. However, there are relatively few studies on whether ferroptosis is a new therapeutic target of hypotensive drugs in the treatment of DKD.

Entresto, also known as LCZ696, is composed of sacubitril and valsartan and has been approved for treating heart failure in clinic^175^. An increasing number of clinical trials have proven that Entresto is favorable for renal protection and reducing hypertension. A recent report demonstrated that Entresto could inhibit ferroptosis to reduce doxorubicin-induced cardiotoxicity. Meanwhile, this finding revealed it could suppress ferroptosis by activating the SIRT3 pathway to reduce myocardial damage, which provided a new perspective for the study of Entresto in DKD^176^. Captopril, a classic anti-hypertensive drug belonging to ACEIs, has been applied in DKD patients to reduce urinary albumin for many years^177^. According to existing studies, captopril seems unable to regulate ferroptosis. A study showed that in a murine model of acute radiation syndrome, iron deposition in the spleen was not mitigated, and the expression level of SLC7A11 remained unchanged after captopril treatment^178^. However, whether ACEIs might regulate ferroptosis in other ways remains unclear. Moreover, calcium channel blockers can mediate iron overload via divalent metal transporter-1^179^, but the availability of calcium channel blockers in suppressing ferroptosis is still unknown. Therefore, more evidence needs to be provided to deeply investigate the key role of antihypertensive drugs in regulating ferroptosis.

### Traditional Chinese medicine components

In addition to the new discoveries of clinical pills, the designation of new therapeutic strategies is indispensable. Traditional Chinese medicines have shown protective effects, whether in antioxidation or anti-inflammation, so they possess huge potential for slowing the development of DKD. Recent studies have found many traditional Chinese herb components have a beneficial effect in suppressing ferroptotic cell death in DKD.

Germacrone, the main bioactive component of *Rhizoma Curcuma*, was found to improve ferroptosis-induced kidney injury in *db/db* mice via the ROS-GSH-GPX4 axis. In MPC5 cells cultured with high glucose, inhibition of ferroptosis alleviated high glucose-induced podocyte mitochondrial damage, and mmu_circRNA_0000309 played an important role in this process^152^. Quercetin, a flavonoid widely distributed in nature, has been verified to decrease the risk of type 2 DM according to epidemiological investigations^180^, and its mechanism is extensive, including antioxidant, anti-inflammatory and anti-fibrotic effects. Recently, Li et al. found that quercetin could suppress ferroptosis of islet β-cells in the development of type 2 DM^181^. At the same time, Jiang et al. found that it could reduce liver lipotoxicity in HFD-induced mice by downregulating mitochondrial ROS-mediated ferroptosis^182^. In addition, quercetin could inhibit ferroptosis and act a protective role in AKI by reducing the expression of activating transcription factor 3 (ATF3)^74^. However, the function of quercetin in DKD-induced ferroptosis requires more experiments to detect. Salidroside, a component of the traditional Chinese medicine *Rhodiola*, was proven to reshape the gut microbiota^183^ and limit the accumulation of iron^184^. In *db/db* mice, it was found that salidroside could reduce blood glucose levels, which could be regulated by changing gut microbiota and regulating iron metabolism^185^. Calycosin (C_16_H_12_O_5_), an isoflavone extracted from the root stem of *Astragali Radix*, has anti-inflammatory, immunoregulatory, antiviral and antioxidant properties^186^. In *db/db* mice and HK-2 cells cultured with high glucose, calycosin inhibited high glucose-induced ferroptosis by affecting GPX4 and NCOA4 levels^164^. Glabridin is a bioactive component of *licorice*. It has been shown to exert hypoglycemic and protective effects on DM and its complications^187^. Tan et al. found that glabridin could restore the antioxidant system damaged by high glucose by decreasing the iron content in the kidney of DKD and recovering the level of ferroptosis-related markers. They proposed that the anti-ferroptosis effect of glabridin was probably mediated through the vascular endothelial growth factor (VEGF)/protein kinase B (Akt)/extracellular regulated protein kinase (ERK) pathway^162^. Platycodin D, extracted from the root of *Platycodon grandiflorum*, is a triterpenoid saponin with multiple pharmacological properties, including antitumor, antiviral, anti-inflammatory, antiaging and neuroprotective effects. It was reported that platycodin D could inhibit high glucose-induced ferroptosis in HK-2 cells by upregulating GPX4^161^. Schisandrin A, one of the lignans isolated from the fruits of *Schisandra chinensis*, is used as a tonic agent in traditional Chinese medicine. It could inhibit ferroptosis and protect against kidney injury by reducing ROS production, mitigating the inflammatory response and alleviating mitochondrial damage through the adiponectin receptor 1 (AdipoR1)/AMPK/Nrf2/HO-1/GPX4 pathway in HFD/STZ-induced diabetic mice^165^. 7-Hydroxycoumarin, also named umbelliferone, is a derivative of coumarin widely present in umbelliferae plants and has many biological activities, such as anti-inflammatory and anti-hyperglycemic activities. It could inhibit ferroptosis by activating the Nrf-2/HO-1 pathway in *db/db* mice and HK-2 cells induced by high glucose, thereby retarding the progression of DKD^166^.

## Conclusion and Outlook

In this review, we introduced the major mechanisms of the development and progression of ferroptosis, including iron overload, decreased antioxidant capacity and lipid peroxides accumulation. Then, we discussed the advances in research on the connection between ferroptosis and DKD and emphasized the role of ferroptotic mediators in DKD. Moreover, we summarized the pharmacological progress of targeting ferroptosis in DKD in the hope of providing new therapeutic strategies for patients with DKD.

To date, accumulating evidence supports the crucial role of ferroptosis in the onset and progression of DKD, and ferroptosis inhibition exerts a protective effect in DKD models^68^, ^88^. However, there are some problems that must be noted. Firstly, the detailed mechanisms that trigger ferroptosis remain unknown. Although deposition of iron in RTECs in DKD patients has been accepted by almost all researchers^78^, does iron overload drive ferroptotic cell death or accumulation of ROS by diverse pathways? What's more, the downstream factors of lipid peroxidation have not been fully clarified. Secondly, more investigations are needed to reveal the role of ferroptosis, especially when multiple pathological conditions coexist in DKD, and whether different factors affect each other. In addition, DKD is mediated by multiple cell death pathways^93^. However, the specific mechanism by which other cell death modes regulate ferroptosis in the development of DKD needs to be further explored. Thirdly, it is important to identify a reliable biomarker for predicting ferroptosis in DKD. The current biomarkers related to ferroptosis used in preclinical studies are nonspecific, such as GPX4 and ferritin^21^, ^33^, which exist in other cell death modes and certain pathological conditions. With the development of proteomic and metabonomic technology, exploring potential biomarkers is more convenient^160^, but subsequent applications in animal experiments and clinical diagnoses require more time and samples^160^. Additionally, as we mentioned above, many hypoglycemic drugs have been proven effective in suppressing ferroptosis, which provides novel strategies for the treatment of ferroptosis-related diseases, such as AKI^188^. Although selective inhibition of ferroptosis has been proven to substantially improve kidney function in various animal models and cell models, clinical trials have not yet been performed with ferroptosis-specific inhibitors to treat DKD. Therefore, the identification of effective ferroptosis-targeted drugs for preventing and curing DKD is extremely necessary and urgent.

Overall, concerning the positive impact of ferroptosis inhibition on the progression of DKD, developing new therapeutic targets is quite valuable. Meanwhile, continuing comprehensive studies on ferroptosis and DKD are indispensable to better understand the key regulatory signaling pathways and underlying potential pathogenesis. Therefore, more in-depth research is required to focus on the role of ferroptosis in the development of DKD, which is of great significance for the clinical diagnosis and treatment of DKD in the future.

## Figures and Tables

**Table 1:** Summary of ferroptosis inducers and inhibitors

Classification	Reagents	Mechanism	Reference
**Ferroptosis Inducers**	Erastin	Inhibition of system Xc^-^ and VDAC2/3	10
	Sulphasalazine	Inhibition of system Xc^-^	10, 48
	Sorafenib	Inhibition of system Xc^-^ and Nrf2	49, 50
	Glutamate	Inhibition of system Xc^-^ and Cys deprivation	51
	RSL3	GPX4 inactivation	32
	Solasonine	GPX4 inactivation	52
	Altretamine	GPX4 inactivation and promotion of ROS	53
	FINO2	GPX4 inactivation and oxidation of unstable iron	54
	FIN56	GPX4 inactivation and CoQ10 deletion	55
	BSO	GSH deletion	32
	DPI2	GSH deletion	32
	Cisplatin	GSH deletion and GPX4 inactivation	56
	Artesunate	Degradation of ferritin and increased cellular iron	57
	iFSP	Inhibition of FSP1	34
	Brequinar	Inhibition of DHODH	36
**Ferroptosis Inhibitors**	Ferrostatin-1	Inhibition of lipid peroxidation	58
	Liproxstatin-1	Inhibition of lipid peroxidation	33
	Necrostatin-1	Inhibition of lipid peroxidation	33, 59
	SRS 16-86	Inhibition of lipid peroxidation	60
	SRS 11-92	Inhibition of lipid peroxidation	58
	Nuciferine	Inhibition of lipid peroxidation	61
	TEMPO	Elimination of ROS	62
	Phenothiazine	Elimination of ROS	63
	Phenoxazine	Elimination of ROS	63
	Diarylamine	Elimination of ROS	63
	Vitamin E	Elimination of ROS and inactivation of LOXs	27, 64
	NDGA	Inactivation of LOXs	65
	Zileuoton	Inactivation of LOXs	66
	Baicalein	Inactivation of LOXs and ACSL4	65, 67
	Thiazolidinedione	Inactivation of ACSL4	68
	Dexrazoxane	Depletion of cellular iron	69
	Deferoxamine	Depletion of cellular iron	69
	Deferiprone	Depletion of cellular iron	70
	β-Mercaptoethanol	Increased cysteine intake	71
	Vitamin K	Activation of FSP1	72
	Curcumin	Activation of Nrf2	73
	Quercetin	Activation of Nrf2 and elimination of ROS	74, 75
	MCTR1	Activation of Nrf2	76
	Irisin	Activation of Nrf2	77

**Table 2:** The associated treatment progress of DKD by targeting ferroptosis

Treatments	Related signaling pathways	Mechanisms	Experimental models	References
**N.A.**	N.A.	Decrease in SLC7A11 and GPX4 expression	DKD patient kidney tubules	83
**N.A.**	N.A.	Increase in ferritin, LDH, ACSL4, PTGS2 and NOX1 expression; Decrease in GPX4 expression	DKD patient serum	86
**Ferroastatin-1**	Upregulate p53 and nuclear Nrf2 expression	Increase in SLC7A11 and GPX4 expression; Decrease in FTH1 expression	STZ-induced diabetic mice; *db/db* mice; TGF-β1-stimulated NRK-52E cells	83
**Ferroastatin-1**	Downregulate HIF-1α/HO-1 pathway	Increase in GPX4, GSH-Px, CAT and SOD expression; Decrease in ferritin, transferrin, MDA and ROS expression	*db/db* mice	136
**Ferroastatin-1**	N.A.	Increase in GPX4 expression; Decrease in ZIP14 expression	STZ-induced diabetic rats; High glucose-treated HK-2 cells	145
**Deferoxamine**	N.A.	Increased in GPX4, FTH1 and SLC7A11 expression; Decreased in ACSL4 and TFR1 expression	High glucose-treated HK-2 cells	161
**Rosiglitazone**	Downregulate IL-6 and TNF-α expression	Increase in GPX4 expression; Decrease in PTGS2 and ASCL4 expression	STZ-induced diabetic mice; *db/db* mice; High glucose-treated NRK-52E cells; High glucose-treated HK-2 cells	68
**Rosiglitazone**	Regulate VEGF/Akt/ERK pathway	Increase in GPX4, SLC7A11 and SLC3A2 expression; Decrease in TFR1 expression	STZ- and HFD- induced diabetic mice; High glucose-treated NRK-52E cells	162
**Fenofibrate**	Upregulate Nrf2 expression	Increase in GPX4, SLC7A11 and FTH1 expression; Decrease in TFR1 expression	STZ-induced diabetic mice; High glucose-treated HK-2 cells	88
**Dapagliflozin**	Upregulate FPN expression	Increase in GPX4 and ASCL4 expression	STZ- and HFD- induced diabetic mice; High glucose-treated HK-2 cells	163
**Calycosin.**	N.A.	Increased in GPX4 expression; Decreased in NCOA4 expression	*db/db* mice; High glucose-treated HK-2 cells	164
**Glabridin**	Regulate VEGF/Akt/ERK pathway	Increase in GPX4, SLC7A11 and SLC3A2 expression; Decrease in TFR1 expression	STZ- and HFD- induced diabetic mice; High glucose-treated NRK-52E cells	162
**Platycodin D**	N.A.	Increased in GPX4, FTH1 and SLC7A11expression; Decreased in ACSL4 and TFR1 expression	High glucose-treated HK-2 cells	161
**Schisandrin A**	Upregulate AdipoR1/AMPK pathway; Downregulate TXNIP/NLRP3 pathway	Increased in GPX4, SOD, CAT and GSH-Px expression; Decreased in LDH expression	STZ- and HFD- induced diabetic mice; High glucose-treated HRGEnCs	165
**Umbelliferone**	Upregulate Nrf2/HO-1 pathway	Increase in GPX4 expression; Decrease in ACSL4 expression	High glucose-treated HK-2 cells; *db/db* mice	166
**NAC combined with insulin**	Upregulate SIRT3 expression	Increase in GPX4 and SLC7A11 expression; Decrease in ferritin and TFR1 expression and the acetylation level of SOD2	STZ- and ALX- induced diabetic Beagles; High glucose-treated MDCK cells	149
**Germacrone**	Regulate mmu_circRNA_0000309/miR-188-3p pathway	Increase in GPX4 and FTH1 expression; Decrease in COX4, ACSL4 and NOX1 expression	*db/db* mice; High glucose-treated MPC5	152
**Overexpression of Sp1**	Upregulate Prdx6 expression	Increase in SLC7A11 and GPX4 expression	STZ-induced diabetic mice; High glucose-treated MPC5	84
**Knockdown of Salusin‑β**	Upregulate Nrf2 expression	Increase in GPX4, SLC7A11 and FTH1 expression; Decreased TFR1 expression	High glucose-treated HK‑2 cells	157
**Knockdown of HMGB1**	Downregulate TLR4/NF-κB pathway Upregulate Nrf2 expression	Increase in GPX4 expression; Decrease in ferritin, LDH, ACSL4, PTGS2 and NOX1 expression	High glucose-treated SV40-MES13 cells	86
**Knockdown of Circ_ASAP2**	Regulate miR‑770‑5p/SOX2/SLC7A11 pathway	Increase in MDA expression; Decrease in GPX4, SOD, CAT and GSH-Px expression	STZ- and HFD- induced diabetic mice; High glucose-treated HK-2 cells	153

**N.A.**: Not applicable.

## Abbreviations

DKD: Diabetic kidney disease
AKI: Acute kidney injury
ROS: Reactive oxygen species
TFR1: Transferrin receptor 1
FTH1: Ferritin heavy chain
LOXs: Lipoxygenases
NCOA4: Nuclear receptor coactivator 4
RTECs: Renal tubular epithelial cells
AA: Arachidonic acid
PUFAs: Polyunsaturated fatty acids
ACSL4: Acyl-CoA synthetase long chain family member 4
NADPH: Nicotinamide adenine dinucleotide phosphate
System Xc^-^: Cystine/glutamate antiporter system
GSH: Glutathione
GPX4: Glutathione peroxidase 4
FSP1: Ferroptosis suppressor protein 1
Nrf2: Nuclear factor erythroid 2-related factor 2
AMPK: AMP-activated protein kinase
ALOX: Arachidonate lipoxygenase
TGF-β1: Transforming growth factor beta 1
NF-κB: Nuclear factor-κB
VDACs: Voltage-dependent anion channel 2/3
HO-1: Heme oxygenase-1
HMGB1: High mobility group 1
FPN: Ferroportin
IRI: ischemia-reperfusion injury
N.A.: Not applicable

## References

[1] Whiting DR, Guariguata L, Weil C, Shaw J (2011). IDF diabetes atlas: global estimates of the prevalence of diabetes for 2011 and 2030. Diabetes Res Clin Pract.

[2] Umanath K, Lewis JB (2018). Update on Diabetic Nephropathy: Core Curriculum 2018. Am J Kidney Dis.

[3] Selby NM, Taal MW (2020). An updated overview of diabetic nephropathy: Diagnosis, prognosis, treatment goals and latest guidelines. Diabetes Obes Metab.

[4] Ricciardi CA, Gnudi L (2021). Kidney disease in diabetes: From mechanisms to clinical presentation and treatment strategies. Metabolism.

[5] Ambinathan JPN, Sridhar VS, Lytvyn Y, Lovblom LE, Liu H, Bjornstad P (2021). Relationships between inflammation, hemodynamic function and RAAS in longstanding type 1 diabetes and diabetic kidney disease. J Diabetes Complications.

[6] Feng Q, Liu D, Lu Y, Liu Z (2020). The Interplay of Renin-Angiotensin System and Toll-Like Receptor 4 in the Inflammation of Diabetic Nephropathy. J Immunol Res.

[7] Gonzalez CD, Carro Negueruela MP, Nicora Santamarina C, Resnik R, Vaccaro MI (2021). Autophagy Dysregulation in Diabetic Kidney Disease: From Pathophysiology to Pharmacological Interventions. Cells.

[8] Wu Y, Chen Y (2022). Research progress on ferroptosis in diabetic kidney disease. Front Endocrinol (Lausanne).

[9] Sanchez-Nino MD, Benito-Martin A, Ortiz A (2010). New paradigms in cell death in human diabetic nephropathy. Kidney Int.

[10] Dixon SJ, Lemberg KM, Lamprecht MR, Skouta R, Zaitsev EM, Gleason CE (2012). Ferroptosis: an iron-dependent form of nonapoptotic cell death. Cell.

[11] Stockwell BR, Friedmann Angeli JP, Bayir H, Bush AI, Conrad M, Dixon SJ (2017). Ferroptosis: A Regulated Cell Death Nexus Linking Metabolism, Redox Biology, and Disease. Cell.

[12] Jiang X, Stockwell BR, Conrad M (2021). Ferroptosis: mechanisms, biology and role in disease. Nat Rev Mol Cell Biol.

[13] Mou Y, Wang J, Wu J, He D, Zhang C, Duan C (2019). Ferroptosis, a new form of cell death: opportunities and challenges in cancer. J Hematol Oncol.

[14] Wei Z, Xie Y, Wei M, Zhao H, Ren K, Feng Q (2022). New insights in ferroptosis: Potential therapeutic targets for the treatment of ischemic stroke. Front Pharmacol.

[15] Feng Q, Yu X, Qiao Y, Pan S, Wang R, Zheng B (2022). Ferroptosis and Acute Kidney Injury (AKI): Molecular Mechanisms and Therapeutic Potentials. Front Pharmacol.

[16] Zhang X, Li LX, Ding H, Torres VE, Yu C, Li X (2021). Ferroptosis Promotes Cyst Growth in Autosomal Dominant Polycystic Kidney Disease Mouse Models. J Am Soc Nephrol.

[17] Wang J, Liu Y, Wang Y, Sun L (2021). The Cross-Link between Ferroptosis and Kidney Diseases. Oxid Med Cell Longev.

[18] Xie Y, Hou W, Song X, Yu Y, Huang J, Sun X (2016). Ferroptosis: process and function. Cell Death Differ.

[19] Harrison PM, Arosio P (1996). The ferritins: molecular properties, iron storage function and cellular regulation. Biochim Biophys Acta.

[20] Valko M, Morris H, Cronin MT (2005). Metals, toxicity and oxidative stress. Curr Med Chem.

[21] Latunde-Dada GO (2017). Ferroptosis: Role of lipid peroxidation, iron and ferritinophagy. Biochim Biophys Acta Gen Subj.

[22] Tang D, Kroemer G (2020). Ferroptosis. Curr Biol.

[23] Song Q, Liao W, Chen X, He Z, Li D, Li B (2021). Oxalate Activates Autophagy to Induce Ferroptosis of Renal Tubular Epithelial Cells and Participates in the Formation of Kidney Stones. Oxid Med Cell Longev.

[24] Kagan VE, Mao G, Qu F, Angeli JP, Doll S, Croix CS (2017). Oxidized arachidonic and adrenic PEs navigate cells to ferroptosis. Nat Chem Biol.

[25] Yang XD, Yang YY (2022). Ferroptosis as a Novel Therapeutic Target for Diabetes and Its Complications. Front Endocrinol (Lausanne).

[26] Doll S, Proneth B, Tyurina YY, Panzilius E, Kobayashi S, Ingold I (2017). ACSL4 dictates ferroptosis sensitivity by shaping cellular lipid composition. Nat Chem Biol.

[27] Shah R, Shchepinov MS, Pratt DA (2018). Resolving the Role of Lipoxygenases in the Initiation and Execution of Ferroptosis. ACS Cent Sci.

[28] Zou Y, Li H, Graham ET, Deik AA, Eaton JK, Wang W (2020). Cytochrome P450 oxidoreductase contributes to phospholipid peroxidation in ferroptosis. Nat Chem Biol.

[29] Yan B, Ai Y, Sun Q, Ma Y, Cao Y, Wang J (2021). Membrane Damage during Ferroptosis Is Caused by Oxidation of Phospholipids Catalyzed by the Oxidoreductases POR and CYB5R1. Mol Cell.

[30] Pandey AV, Fluck CE (2013). NADPH P450 oxidoreductase: structure, function, and pathology of diseases. Pharmacol Ther.

[31] Conrad M, Friedmann Angeli JP (2015). Glutathione peroxidase 4 (Gpx4) and ferroptosis: what's so special about it?. Mol Cell Oncol.

[32] Yang WS, SriRamaratnam R, Welsch ME, Shimada K, Skouta R, Viswanathan VS (2014). Regulation of ferroptotic cancer cell death by GPX4. Cell.

[33] Friedmann Angeli JP, Schneider M, Proneth B, Tyurina YY, Tyurin VA, Hammond VJ (2014). Inactivation of the ferroptosis regulator Gpx4 triggers acute renal failure in mice. Nat Cell Biol.

[34] Doll S, Freitas FP, Shah R, Aldrovandi M, da Silva MC, Ingold I (2019). FSP1 is a glutathione-independent ferroptosis suppressor. Nature.

[35] Kraft VAN, Bezjian CT, Pfeiffer S, Ringelstetter L, Muller C, Zandkarimi F (2020). GTP Cyclohydrolase 1/Tetrahydrobiopterin Counteract Ferroptosis through Lipid Remodeling. ACS Cent Sci.

[36] Mao C, Liu X, Zhang Y, Lei G, Yan Y, Lee H (2021). DHODH-mediated ferroptosis defence is a targetable vulnerability in cancer. Nature.

[37] Ji H, Wang W, Li X, Han X, Zhang X, Wang J (2022). p53: A double-edged sword in tumor ferroptosis. Pharmacol Res.

[38] Lee H, Zandkarimi F, Zhang Y, Meena JK, Kim J, Zhuang L (2020). Energy-stress-mediated AMPK activation inhibits ferroptosis. Nat Cell Biol.

[39] Dodson M, Castro-Portuguez R, Zhang DD (2019). NRF2 plays a critical role in mitigating lipid peroxidation and ferroptosis. Redox Biol.

[40] Jiang L, Kon N, Li T, Wang SJ, Su T, Hibshoosh H (2015). Ferroptosis as a p53-mediated activity during tumour suppression. Nature.

[41] Wang Y, Yang L, Zhang X, Cui W, Liu Y, Sun QR (2019). Epigenetic regulation of ferroptosis by H2B monoubiquitination and p53. EMBO Rep.

[42] Ou Y, Wang SJ, Li D, Chu B, Gu W (2016). Activation of SAT1 engages polyamine metabolism with p53-mediated ferroptotic responses. Proc Natl Acad Sci U S A.

[43] Chu B, Kon N, Chen D, Li T, Liu T, Jiang L (2019). ALOX12 is required for p53-mediated tumour suppression through a distinct ferroptosis pathway. Nat Cell Biol.

[44] Jennis M, Kung CP, Basu S, Budina-Kolomets A, Leu JI, Khaku S (2016). An African-specific polymorphism in the TP53 gene impairs p53 tumor suppressor function in a mouse model. Genes Dev.

[45] Liu J, Zhang C, Wang J, Hu W, Feng Z (2020). The Regulation of Ferroptosis by Tumor Suppressor p53 and its Pathway. Int J Mol Sci.

[46] Xie Y, Zhu S, Song X, Sun X, Fan Y, Liu J (2017). The Tumor Suppressor p53 Limits Ferroptosis by Blocking DPP4 Activity. Cell Rep.

[47] Tarangelo A, Magtanong L, Bieging-Rolett KT, Li Y, Ye J, Attardi LD (2018). p53 Suppresses Metabolic Stress-Induced Ferroptosis in Cancer Cells. Cell Rep.

[48] Zhuang J, Liu X, Yang Y, Zhang Y, Guan G (2021). Sulfasalazine, a potent suppressor of gastric cancer proliferation and metastasis by inhibition of xCT: Conventional drug in new use. J Cell Mol Med.

[49] Louandre C, Ezzoukhry Z, Godin C, Barbare JC, Maziere JC, Chauffert B (2013). Iron-dependent cell death of hepatocellular carcinoma cells exposed to sorafenib. Int J Cancer.

[50] Sun X, Ou Z, Chen R, Niu X, Chen D, Kang R (2016). Activation of the p62-Keap1-NRF2 pathway protects against ferroptosis in hepatocellular carcinoma cells. Hepatology.

[51] Zhang X, Yu K, Ma L, Qian Z, Tian X, Miao Y (2021). Endogenous glutamate determines ferroptosis sensitivity via ADCY10-dependent YAP suppression in lung adenocarcinoma. Theranostics.

[52] Jin M, Shi C, Li T, Wu Y, Hu C, Huang G (2020). Solasonine promotes ferroptosis of hepatoma carcinoma cells via glutathione peroxidase 4-induced destruction of the glutathione redox system. Biomed Pharmacother.

[53] Woo JH, Shimoni Y, Yang WS, Subramaniam P, Iyer A, Nicoletti P (2015). Elucidating Compound Mechanism of Action by Network Perturbation Analysis. Cell.

[54] Gaschler MM, Andia AA, Liu H, Csuka JM, Hurlocker B, Vaiana CA (2018). FINO(2) initiates ferroptosis through GPX4 inactivation and iron oxidation. Nat Chem Biol.

[55] Shimada K, Skouta R, Kaplan A, Yang WS, Hayano M, Dixon SJ (2016). Global survey of cell death mechanisms reveals metabolic regulation of ferroptosis. Nat Chem Biol.

[56] Yamaguchi H, Hsu JL, Chen CT, Wang YN, Hsu MC, Chang SS (2013). Caspase-independent cell death is involved in the negative effect of EGF receptor inhibitors on cisplatin in non-small cell lung cancer cells. Clin Cancer Res.

[57] Yang ND, Tan SH, Ng S, Shi Y, Zhou J, Tan KS (2014). Artesunate induces cell death in human cancer cells via enhancing lysosomal function and lysosomal degradation of ferritin. J Biol Chem.

[58] Skouta R, Dixon SJ, Wang J, Dunn DE, Orman M, Shimada K (2014). Ferrostatins inhibit oxidative lipid damage and cell death in diverse disease models. J Am Chem Soc.

[59] Yuk H, Abdullah M, Kim DH, Lee H, Lee SJ (2021). Necrostatin-1 Prevents Ferroptosis in a RIPK1- and IDO-Independent Manner in Hepatocellular Carcinoma. Antioxidants (Basel).

[60] Linkermann A, Stockwell BR, Krautwald S, Anders HJ (2014). Regulated cell death and inflammation: an auto-amplification loop causes organ failure. Nat Rev Immunol.

[61] Li D, Liu B, Fan Y, Liu M, Han B, Meng Y (2021). Nuciferine protects against folic acid-induced acute kidney injury by inhibiting ferroptosis. Br J Pharmacol.

[62] Griesser M, Shah R, Van Kessel AT, Zilka O, Haidasz EA, Pratt DA (2018). The Catalytic Reaction of Nitroxides with Peroxyl Radicals and Its Relevance to Their Cytoprotective Properties. J Am Chem Soc.

[63] Shah R, Margison K, Pratt DA (2017). The Potency of Diarylamine Radical-Trapping Antioxidants as Inhibitors of Ferroptosis Underscores the Role of Autoxidation in the Mechanism of Cell Death. ACS Chem Biol.

[64] Hinman A, Holst CR, Latham JC, Bruegger JJ, Ulas G, McCusker KP (2018). Vitamin E hydroquinone is an endogenous regulator of ferroptosis via redox control of 15-lipoxygenase. PLoS One.

[65] Probst L, Dachert J, Schenk B, Fulda S (2017). Lipoxygenase inhibitors protect acute lymphoblastic leukemia cells from ferroptotic cell death. Biochem Pharmacol.

[66] Yang WS, Stockwell BR (2016). Ferroptosis: Death by Lipid Peroxidation. Trends Cell Biol.

[67] Li M, Meng Z, Yu S, Li J, Wang Y, Yang W (2022). Baicalein ameliorates cerebral ischemia-reperfusion injury by inhibiting ferroptosis via regulating GPX4/ACSL4/ACSL3 axis. Chem Biol Interact.

[68] Wang Y, Bi R, Quan F, Cao Q, Lin Y, Yue C (2020). Ferroptosis involves in renal tubular cell death in diabetic nephropathy. Eur J Pharmacol.

[69] Fernandez-Garcia V, Gonzalez-Ramos S, Martin-Sanz P, Castrillo A, Bosca L (2022). Unraveling the interplay between iron homeostasis, ferroptosis and extramedullary hematopoiesis. Pharmacol Res.

[70] Masaldan S, Clatworthy SAS, Gamell C, Meggyesy PM, Rigopoulos AT, Haupt S (2018). Iron accumulation in senescent cells is coupled with impaired ferritinophagy and inhibition of ferroptosis. Redox Biol.

[71] Chen X, Li J, Kang R, Klionsky DJ, Tang D (2021). Ferroptosis: machinery and regulation. Autophagy.

[72] Mishima E, Ito J, Wu Z, Nakamura T, Wahida A, Doll S (2022). A non-canonical vitamin K cycle is a potent ferroptosis suppressor. Nature.

[73] Yang C, Han M, Li R, Zhou L, Zhang Y, Duan L (2021). Curcumin Nanoparticles Inhibiting Ferroptosis for the Enhanced Treatment of Intracerebral Hemorrhage. Int J Nanomedicine.

[74] Wang Y, Quan F, Cao Q, Lin Y, Yue C, Bi R (2021). Quercetin alleviates acute kidney injury by inhibiting ferroptosis. J Adv Res.

[75] Feng Q, Yang Y, Qiao Y, Zheng Y, Yu X, Liu F (2023). Quercetin Ameliorates Diabetic Kidney Injury by Inhibiting Ferroptosis via Activating Nrf2/HO-1 Signaling Pathway. Am J Chin Med.

[76] Ye J, Peng J, Liu K, Zhang T, Huang W (2022). MCTR1 inhibits ferroptosis by promoting NRF2 expression to attenuate hepatic ischemia-reperfusion injury. Am J Physiol Gastrointest Liver Physiol.

[77] Wang J, Zhu Q, Wang Y, Peng J, Shao L, Li X (2022). Irisin protects against sepsis-associated encephalopathy by suppressing ferroptosis via activation of the Nrf2/GPX4 signal axis. Free Radic Biol Med.

[78] Dominguez JH, Liu Y, Kelly KJ (2015). Renal iron overload in rats with diabetic nephropathy. Physiol Rep.

[79] Howard RL, Buddington B, Alfrey AC (1991). Urinary albumin, transferrin and iron excretion in diabetic patients. Kidney Int.

[80] Chaudhary K, Chilakala A, Ananth S, Mandala A, Veeranan-Karmegam R, Powell FL (2019). Renal iron accelerates the progression of diabetic nephropathy in the HFE gene knockout mouse model of iron overload. Am J Physiol Renal Physiol.

[81] Matsumoto M, Sasaki N, Tsujino T, Akahori H, Naito Y, Masuyama T (2013). Iron restriction prevents diabetic nephropathy in Otsuka Long-Evans Tokushima fatty rat. Ren Fail.

[82] Zhao L, Zou Y, Zhang J, Zhang R, Ren H, Li L (2020). Serum transferrin predicts end-stage Renal Disease in Type 2 Diabetes Mellitus patients. Int J Med Sci.

[83] Kim S, Kang SW, Joo J, Han SH, Shin H, Nam BY (2021). Characterization of ferroptosis in kidney tubular cell death under diabetic conditions. Cell Death Dis.

[84] Zhang Q, Hu Y, Hu JE, Ding Y, Shen Y, Xu H (2021). Sp1-mediated upregulation of Prdx6 expression prevents podocyte injury in diabetic nephropathy via mitigation of oxidative stress and ferroptosis. Life Sci.

[85] Wu WY, Wang ZX, Li TS, Ding XQ, Liu ZH, Yang J (2022). SSBP1 drives high fructose-induced glomerular podocyte ferroptosis via activating DNA-PK/p53 pathway. Redox Biol.

[86] Wu Y, Zhao Y, Yang HZ, Wang YJ, Chen Y (2021). HMGB1 regulates ferroptosis through Nrf2 pathway in mesangial cells in response to high glucose. Biosci Rep.

[87] Luo EF, Li HX, Qin YH, Qiao Y, Yan GL, Yao YY (2021). Role of ferroptosis in the process of diabetes-induced endothelial dysfunction. World J Diabetes.

[88] Li S, Zheng L, Zhang J, Liu X, Wu Z (2021). Inhibition of ferroptosis by up-regulating Nrf2 delayed the progression of diabetic nephropathy. Free Radic Biol Med.

[89] Jha JC, Banal C, Chow BS, Cooper ME, Jandeleit-Dahm K (2016). Diabetes and Kidney Disease: Role of Oxidative Stress. Antioxid Redox Signal.

[90] Jha JC, Dai A, Garzarella J, Charlton A, Urner S, Ostergaard JA (2022). Independent of Renox, NOX5 Promotes Renal Inflammation and Fibrosis in Diabetes by Activating ROS-Sensitive Pathways. Diabetes.

[91] Stitt-Cavanagh E, MacLeod L, Kennedy C (2009). The podocyte in diabetic kidney disease. ScientificWorldJournal.

[92] Casalena GA, Yu L, Gil R, Rodriguez S, Sosa S, Janssen W (2020). The diabetic microenvironment causes mitochondrial oxidative stress in glomerular endothelial cells and pathological crosstalk with podocytes. Cell Commun Signal.

[93] Park E, Chung SW (2019). ROS-mediated autophagy increases intracellular iron levels and ferroptosis by ferritin and transferrin receptor regulation. Cell Death Dis.

[94] Wang YH, Chang DY, Zhao MH, Chen M (2022). Glutathione Peroxidase 4 Is a Predictor of Diabetic Kidney Disease Progression in Type 2 Diabetes Mellitus. Oxid Med Cell Longev.

[95] Li Z, Deng H, Guo X, Yan S, Lu C, Zhao Z (2022). Effective dose/duration of natural flavonoid quercetin for treatment of diabetic nephropathy: A systematic review and meta-analysis of rodent data. Phytomedicine.

[96] Hayashi D, Yagi K, Song C, Ueda S, Yamanoue M, Topham M (2017). Diacylglycerol Kinase alpha is Involved in the Vitamin E-Induced Amelioration of Diabetic Nephropathy in Mice. Sci Rep.

[97] Rayego-Mateos S, Morgado-Pascual JL, Opazo-Rios L, Guerrero-Hue M, Garcia-Caballero C, Vazquez-Carballo C (2020). Pathogenic Pathways and Therapeutic Approaches Targeting Inflammation in Diabetic Nephropathy. Int J Mol Sci.

[98] Navarro-Gonzalez JF, Mora-Fernandez C (2008). The role of inflammatory cytokines in diabetic nephropathy. J Am Soc Nephrol.

[99] Wang Y, Zhu X, Yuan S, Wen S, Liu X, Wang C (2019). TLR4/NF-kappaB Signaling Induces GSDMD-Related Pyroptosis in Tubular Cells in Diabetic Kidney Disease. Front Endocrinol (Lausanne).

[100] Chen D, Liu Y, Chen J, Lin H, Guo H, Wu Y (2021). JAK/STAT pathway promotes the progression of diabetic kidney disease via autophagy in podocytes. Eur J Pharmacol.

[101] Du N, Liu S, Cui C, Hao F, Gao M, Xu Z (2020). Yishenhuoxue formula regulates TGF-beta/Smad signal transduction to protect rats against Diabetic kidney disease injury. Pak J Pharm Sci.

[102] Araujo AC, Wheelock CE, Haeggstrom JZ (2018). The Eicosanoids, Redox-Regulated Lipid Mediators in Immunometabolic Disorders. Antioxid Redox Signal.

[103] Li C, Deng X, Xie X, Liu Y, Friedmann Angeli JP, Lai L (2018). Activation of Glutathione Peroxidase 4 as a Novel Anti-inflammatory Strategy. Front Pharmacol.

[104] Thimmulappa RK, Lee H, Rangasamy T, Reddy SP, Yamamoto M, Kensler TW (2006). Nrf2 is a critical regulator of the innate immune response and survival during experimental sepsis. J Clin Invest.

[105] Kobayashi EH, Suzuki T, Funayama R, Nagashima T, Hayashi M, Sekine H (2016). Nrf2 suppresses macrophage inflammatory response by blocking proinflammatory cytokine transcription. Nat Commun.

[106] Linkermann A, Skouta R, Himmerkus N, Mulay SR, Dewitz C, De Zen F (2014). Synchronized renal tubular cell death involves ferroptosis. Proc Natl Acad Sci U S A.

[107] Splichal I, Donovan SM, Jenistova V, Splichalova I, Salmonova H, Vlkova E (2019). High Mobility Group Box 1 and TLR4 Signaling Pathway in Gnotobiotic Piglets Colonized/Infected with L. amylovorus, L. mucosae, E. coli Nissle 1917 and S. Typhimurium. Int J Mol Sci.

[108] Han F, Li S, Yang Y, Bai Z (2021). Interleukin-6 promotes ferroptosis in bronchial epithelial cells by inducing reactive oxygen species-dependent lipid peroxidation and disrupting iron homeostasis. Bioengineered.

[109] Liu Y, Zhou L, Lv C, Liu L, Miao S, Xu Y (2023). PGE2 pathway mediates oxidative stress-induced ferroptosis in renal tubular epithelial cells. FEBS J.

[110] Proneth B, Conrad M (2019). Ferroptosis and necroinflammation, a yet poorly explored link. Cell Death Differ.

[111] Langmesser S, Albrecht U (2006). Life time-circadian clocks, mitochondria and metabolism. Chronobiol Int.

[112] Ahmad AA, Draves SO, Rosca M (2021). Mitochondria in Diabetic Kidney Disease. Cells.

[113] Cargill K, Sims-Lucas S (2020). Metabolic requirements of the nephron. Pediatr Nephrol.

[114] Colombini M (2012). VDAC structure, selectivity, and dynamics. Biochim Biophys Acta.

[115] DeHart DN, Fang D, Heslop K, Li L, Lemasters JJ, Maldonado EN (2018). Opening of voltage dependent anion channels promotes reactive oxygen species generation, mitochondrial dysfunction and cell death in cancer cells. Biochem Pharmacol.

[116] Gao M, Yi J, Zhu J, Minikes AM, Monian P, Thompson CB (2019). Role of Mitochondria in Ferroptosis. Mol Cell.

[117] Bernardi P, Di Lisa F (2015). The mitochondrial permeability transition pore: molecular nature and role as a target in cardioprotection. J Mol Cell Cardiol.

[118] Novgorodov SA, Voltin JR, Gooz MA, Li L, Lemasters JJ, Gudz TI (2018). Acid sphingomyelinase promotes mitochondrial dysfunction due to glutamate-induced regulated necrosis. J Lipid Res.

[119] Maher P, van Leyen K, Dey PN, Honrath B, Dolga A, Methner A (2018). The role of Ca(2+) in cell death caused by oxidative glutamate toxicity and ferroptosis. Cell Calcium.

[120] Wang X, Lu S, He C, Wang C, Wang L, Piao M (2019). RSL3 induced autophagic death in glioma cells via causing glycolysis dysfunction. Biochem Biophys Res Commun.

[121] Ma X, Xiao L, Liu L, Ye L, Su P, Bi E (2021). CD36-mediated ferroptosis dampens intratumoral CD8(+) T cell effector function and impairs their antitumor ability. Cell Metab.

[122] Gai Z, Wang T, Visentin M, Kullak-Ublick GA, Fu X, Wang Z (2019). Lipid Accumulation and Chronic Kidney Disease. Nutrients.

[123] Feng L, Gu C, Li Y, Huang J (2017). High Glucose Promotes CD36 Expression by Upregulating Peroxisome Proliferator-Activated Receptor gamma Levels to Exacerbate Lipid Deposition in Renal Tubular Cells. Biomed Res Int.

[124] Hou Y, Wu M, Wei J, Ren Y, Du C, Wu H (2015). CD36 is involved in high glucose-induced epithelial to mesenchymal transition in renal tubular epithelial cells. Biochem Biophys Res Commun.

[125] Chan JY, Kwong M (2000). Impaired expression of glutathione synthetic enzyme genes in mice with targeted deletion of the Nrf2 basic-leucine zipper protein. Biochim Biophys Acta.

[126] Chen DQ, Wu J, Li P (2022). Therapeutic mechanism and clinical application of Chinese herbal medicine against diabetic kidney disease. Front Pharmacol.

[127] Zhao S, Ghosh A, Lo CS, Chenier I, Scholey JW, Filep JG (2018). Nrf2 Deficiency Upregulates Intrarenal Angiotensin-Converting Enzyme-2 and Angiotensin 1-7 Receptor Expression and Attenuates Hypertension and Nephropathy in Diabetic Mice. Endocrinology.

[128] Abdalkader M, Lampinen R, Kanninen KM, Malm TM, Liddell JR (2018). Targeting Nrf2 to Suppress Ferroptosis and Mitochondrial Dysfunction in Neurodegeneration. Front Neurosci.

[129] Kerins MJ, Ooi A (2018). The Roles of NRF2 in Modulating Cellular Iron Homeostasis. Antioxid Redox Signal.

[130] Kuang F, Liu J, Tang D, Kang R (2020). Oxidative Damage and Antioxidant Defense in Ferroptosis. Front Cell Dev Biol.

[131] Fujiki K, Inamura H, Sugaya T, Matsuoka M (2019). Blockade of ALK4/5 signaling suppresses cadmium- and erastin-induced cell death in renal proximal tubular epithelial cells via distinct signaling mechanisms. Cell Death Differ.

[132] Tian H, Xiong Y, Zhang Y, Leng Y, Tao J, Li L (2021). Activation of NRF2/FPN1 pathway attenuates myocardial ischemia-reperfusion injury in diabetic rats by regulating iron homeostasis and ferroptosis. Cell Stress Chaperones.

[133] Abraham NG, Cao J, Sacerdoti D, Li X, Drummond G (2009). Heme oxygenase: the key to renal function regulation. Am J Physiol Renal Physiol.

[134] Jiang N, Zhao H, Han Y, Li L, Xiong S, Zeng L (2020). HIF-1alpha ameliorates tubular injury in diabetic nephropathy via HO-1-mediated control of mitochondrial dynamics. Cell Prolif.

[135] Adedoyin O, Boddu R, Traylor A, Lever JM, Bolisetty S, George JF (2018). Heme oxygenase-1 mitigates ferroptosis in renal proximal tubule cells. Am J Physiol Renal Physiol.

[136] Feng X, Wang S, Sun Z, Dong H, Yu H, Huang M (2021). Ferroptosis Enhanced Diabetic Renal Tubular Injury via HIF-1alpha/HO-1 Pathway in db/db Mice. Front Endocrinol (Lausanne).

[137] Gao Z, Zhang Z, Gu D, Li Y, Zhang K, Dong X (2022). Hemin mitigates contrast-induced nephropathy by inhibiting ferroptosis via HO-1/Nrf2/GPX4 pathway. Clin Exp Pharmacol Physiol.

[138] Xu T, Jiang L, Wang Z (2019). The progression of HMGB1-induced autophagy in cancer biology. Onco Targets Ther.

[139] Chen X, Ma J, Kwan T, Stribos EGD, Messchendorp AL, Loh YW (2018). Blockade of HMGB1 Attenuates Diabetic Nephropathy in Mice. Sci Rep.

[140] Ye F, Chai W, Xie M, Yang M, Yu Y, Cao L (2019). HMGB1 regulates erastin-induced ferroptosis via RAS-JNK/p38 signaling in HL-60/NRAS(Q61L) cells. Am J Cancer Res.

[141] van Raaij S, van Swelm R, Bouman K, Cliteur M, van den Heuvel MC, Pertijs J (2018). Tubular iron deposition and iron handling proteins in human healthy kidney and chronic kidney disease. Sci Rep.

[142] van Raaij SEG, Srai SKS, Swinkels DW, van Swelm RPL (2019). Iron uptake by ZIP8 and ZIP14 in human proximal tubular epithelial cells. Biometals.

[143] Song CC, Pantopoulos K, Chen GH, Zhong CC, Zhao T, Zhang DG (2022). Iron increases lipid deposition via oxidative stress-mediated mitochondrial dysfunction and the HIF1alpha-PPARgamma pathway. Cell Mol Life Sci.

[144] Yu Y, Jiang L, Wang H, Shen Z, Cheng Q, Zhang P (2020). Hepatic transferrin plays a role in systemic iron homeostasis and liver ferroptosis. Blood.

[145] Wu K, Fei L, Wang X, Lei Y, Yu L, Xu W (2022). ZIP14 is involved in iron deposition and triggers ferroptosis in diabetic nephropathy. Metallomics.

[146] Atkuri KR, Mantovani JJ, Herzenberg LA, Herzenberg LA (2007). N-Acetylcysteine-a safe antidote for cysteine/glutathione deficiency. Curr Opin Pharmacol.

[147] Nogueira GB, Punaro GR, Oliveira CS, Maciel FR, Fernandes TO, Lima DY (2018). N-acetylcysteine protects against diabetic nephropathy through control of oxidative and nitrosative stress by recovery of nitric oxide in rats. Nitric Oxide.

[148] Wang C, Zhu L, Yuan W, Sun L, Xia Z, Zhang Z (2020). Diabetes aggravates myocardial ischaemia reperfusion injury via activating Nox2-related programmed cell death in an AMPK-dependent manner. J Cell Mol Med.

[149] Li Q, Liao J, Chen W, Zhang K, Li H, Ma F (2022). NAC alleviative ferroptosis in diabetic nephropathy via maintaining mitochondrial redox homeostasis through activating SIRT3-SOD2/Gpx4 pathway. Free Radic Biol Med.

[150] Clark AJ, Parikh SM (2021). Targeting energy pathways in kidney disease: the roles of sirtuins, AMPK, and PGC1alpha. Kidney Int.

[151] Dugan LL, You YH, Ali SS, Diamond-Stanic M, Miyamoto S, DeCleves AE (2013). AMPK dysregulation promotes diabetes-related reduction of superoxide and mitochondrial function. J Clin Invest.

[152] Jin J, Wang Y, Zheng D, Liang M, He Q (2022). A Novel Identified Circular RNA, mmu_mmu_circRNA_0000309, Involves in Germacrone-Mediated Improvement of Diabetic Nephropathy Through Regulating Ferroptosis by Targeting miR-188-3p/GPX4 Signaling Axis. Antioxid Redox Signal.

[153] Li Q, Meng X, Hua Q (2022). Circ ASAP2 decreased inflammation and ferroptosis in diabetic nephropathy through SOX2/SLC7A11 by miR-770-5p. Acta Diabetol.

[154] Lu B, Chen XB, Hong YC, Zhu H, He QJ, Yang B (2019). Identification of PRDX6 as a regulator of ferroptosis. Acta Pharmacol Sin.

[155] Lee DH, Park JH, Han SB, Yoon DY, Jung YY, Hong JT (2017). Peroxiredoxin 6 overexpression attenuates lipopolysaccharide-induced acute kidney injury. Oncotarget.

[156] Shichiri M, Ishimaru S, Ota T, Nishikawa T, Isogai T, Hirata Y (2003). Salusins: newly identified bioactive peptides with hemodynamic and mitogenic activities. Nat Med.

[157] Wang WJ, Jiang X, Gao CC, Chen ZW (2021). Salusinbeta participates in high glucoseinduced HK2 cell ferroptosis in a Nrf2dependent manner. Mol Med Rep.

[158] Zhou LT, Zhang ZJ, Cao JY, Chen H, Zhu YS, Wu X (2021). The unique molecular mechanism of diabetic nephropathy: a bioinformatics analysis of over 250 microarray datasets. Clin Kidney J.

[159] Hu Y, Liu S, Liu W, Zhang Z, Liu Y, Sun D (2021). Bioinformatics analysis of genes related to iron death in diabetic nephropathy through network and pathway levels based approaches. PLoS One.

[160] Luo M, Zhang Z, Lu Y, Feng W, Wu H, Fan L (2022). Urine metabolomics reveals biomarkers and the underlying pathogenesis of diabetic kidney disease. Int Urol Nephrol.

[161] Huang J, Chen G, Wang J, Liu S, Su J (2022). Platycodin D regulates high glucose-induced ferroptosis of HK-2 cells through glutathione peroxidase 4 (GPX4). Bioengineered.

[162] Tan H, Chen J, Li Y, Li Y, Zhong Y, Li G (2022). Glabridin, a bioactive component of licorice, ameliorates diabetic nephropathy by regulating ferroptosis and the VEGF/Akt/ERK pathways. Mol Med.

[163] Huang B, Wen W, Ye S (2022). Dapagliflozin Ameliorates Renal Tubular Ferroptosis in Diabetes via SLC40A1 Stabilization. Oxid Med Cell Longev.

[164] Huang D, Shen P, Wang C, Gao J, Ye C, Wu F (2022). Calycosin plays a protective role in diabetic kidney disease through the regulation of ferroptosis. Pharm Biol.

[165] Wang X, Li Q, Sui B, Xu M, Pu Z, Qiu T (2022). Schisandrin A from Schisandra chinensis Attenuates Ferroptosis and NLRP3 Inflammasome-Mediated Pyroptosis in Diabetic Nephropathy through Mitochondrial Damage by AdipoR1 Ubiquitination. Oxid Med Cell Longev.

[166] Jin T, Chen C (2022). Umbelliferone delays the progression of diabetic nephropathy by inhibiting ferroptosis through activation of the Nrf-2/HO-1 pathway. Food Chem Toxicol.

[167] Kumar S, Prange A, Schulze J, Lettis S, Barnett AH (1998). Troglitazone, an insulin action enhancer, improves glycaemic control and insulin sensitivity in elderly type 2 diabetic patients. Diabet Med.

[168] Kim JH, Lewin TM, Coleman RA (2001). Expression and characterization of recombinant rat Acyl-CoA synthetases 1, 4, and 5. Selective inhibition by triacsin C and thiazolidinediones. J Biol Chem.

[169] Liu Q, Zhang X, Cheng R, Ma JX, Yi J, Li J (2019). Salutary effect of fenofibrate on type 1 diabetic retinopathy via inhibiting oxidative stress-mediated Wnt/beta-catenin pathway activation. Cell Tissue Res.

[170] Forst T, Mathieu C, Giorgino F, Wheeler DC, Papanas N, Schmieder RE (2022). New strategies to improve clinical outcomes for diabetic kidney disease. BMC Med.

[171] Quagliariello V, De Laurentiis M, Rea D, Barbieri A, Monti MG, Carbone A (2021). The SGLT-2 inhibitor empagliflozin improves myocardial strain, reduces cardiac fibrosis and pro-inflammatory cytokines in non-diabetic mice treated with doxorubicin. Cardiovasc Diabetol.

[172] Harder H, Nielsen L, Tu DT, Astrup A (2004). The effect of liraglutide, a long-acting glucagon-like peptide 1 derivative, on glycemic control, body composition, and 24-h energy expenditure in patients with type 2 diabetes. Diabetes Care.

[173] Song JX, An JR, Chen Q, Yang XY, Jia CL, Xu S (2022). Liraglutide attenuates hepatic iron levels and ferroptosis in db/db mice. Bioengineered.

[174] An JR, Su JN, Sun GY, Wang QF, Fan YD, Jiang N (2022). Liraglutide Alleviates Cognitive Deficit in db/db Mice: Involvement in Oxidative Stress, Iron Overload, and Ferroptosis. Neurochem Res.

[175] Li Y, Kang L, Rong K, Zhang Y, Suo Y, Yuan M (2021). Renal protective effects and mechanisms of the angiotensin receptor-neprilysin inhibitor LCZ696 in mice with cardiorenal syndrome. Life Sci.

[176] Liu X, Li D, Pi W, Wang B, Xu S, Yu L (2022). LCZ696 protects against doxorubicin-induced cardiotoxicity by inhibiting ferroptosis via AKT/SIRT3/SOD2 signaling pathway activation. Int Immunopharmacol.

[177] Hsu FY, Lin FJ, Ou HT, Huang SH, Wang CC (2017). Renoprotective Effect of Angiotensin-Converting Enzyme Inhibitors and Angiotensin II Receptor Blockers in Diabetic Patients with Proteinuria. Kidney Blood Press Res.

[178] Rittase WB, Slaven JE, Suzuki YJ, Muir JM, Lee SH, Rusnak M (2022). Iron Deposition and Ferroptosis in the Spleen in a Murine Model of Acute Radiation Syndrome. Int J Mol Sci.

[179] Ludwiczek S, Theurl I, Muckenthaler MU, Jakab M, Mair SM, Theurl M (2007). Ca2+ channel blockers reverse iron overload by a new mechanism via divalent metal transporter-1. Nat Med.

[180] Yao Z, Gu Y, Zhang Q, Liu L, Meng G, Wu H (2019). Estimated daily quercetin intake and association with the prevalence of type 2 diabetes mellitus in Chinese adults. Eur J Nutr.

[181] Li D, Jiang C, Mei G, Zhao Y, Chen L, Liu J (2020). Quercetin Alleviates Ferroptosis of Pancreatic beta Cells in Type 2 Diabetes. Nutrients.

[182] Jiang JJ, Zhang GF, Zheng JY, Sun JH, Ding SB (2022). Targeting Mitochondrial ROS-Mediated Ferroptosis by Quercetin Alleviates High-Fat Diet-Induced Hepatic Lipotoxicity. Front Pharmacol.

[183] Yuan Y, Wu X, Zhang X, Hong Y, Yan H (2019). Ameliorative effect of salidroside from Rhodiola Rosea L. on the gut microbiota subject to furan-induced liver injury in a mouse model. Food Chem Toxicol.

[184] Chen H, Zhu J, Le Y, Pan J, Liu Y, Liu Z (2022). Salidroside inhibits doxorubicin-induced cardiomyopathy by modulating a ferroptosis-dependent pathway. Phytomedicine.

[185] Shi J, Zhao Q, Hao DD, Miao HX, Wan S, Zhou CH (2022). Gut microbiota profiling revealed the regulating effects of salidroside on iron metabolism in diabetic mice. Front Endocrinol (Lausanne).

[186] Nie XH, Ou-yang J, Xing Y, Li DY, Liu RE, Xu RX (2016). Calycosin inhibits migration and invasion through modulation of transforming growth factor beta-mediated mesenchymal properties in U87 and U251 cells. Drug Des Devel Ther.

[187] Wu F, Jin Z, Jin J (2013). Hypoglycemic effects of glabridin, a polyphenolic flavonoid from licorice, in an animal model of diabetes mellitus. Mol Med Rep.

[188] Wang Y, Zhang M, Bi R, Su Y, Quan F, Lin Y (2022). ACSL4 deficiency confers protection against ferroptosis-mediated acute kidney injury. Redox Biol.

